# Notes

**Published:** 1898-06-25

**Authors:** 


### NOTES.

In connection with the Belfast riots a good many cases of injuries and wounds were received at the Royal Hospital, and several mounted police suffering from severe contusions are at present in the wards.

The new Home for Crippled Children at West Bournemouth built by Mr. Passmore Edwards, has just been opened by the Marquis of Northampton. The home overlooking the sea accommodates twenty children, and is suitably built and equipped for its purpose.

A temporary building to accommodate about 95 patients is to be erected down the centre of the garden of The London Hospital, in which the patients who will be disturbed by the raising of the hospital and the improvement of "Cotton" and "Davis" Wards will be placed. Room for the electrical and skiagraphic work of the hospital is also to be provided in this building.

